# Hypoxic conditions affect transcriptome of endometrial stromal cells in endometriosis and promote TGFBI axis

**DOI:** 10.3389/fendo.2024.1465393

**Published:** 2024-12-18

**Authors:** Meruert Sarsenova, Nageswara Rao Boggavarapu, Keiu Kask, Vijayachitra Modhukur, Külli Samuel, Helle Karro, Kristina Gemzell-Danielsson, Parameswaran Grace Luther Lalitkumar, Andres Salumets, Maire Peters, Darja Lavogina

**Affiliations:** ^1^ Department of Obstetrics and Gynaecology, Institute of Clinical Medicine, University of Tartu, Tartu, Estonia; ^2^ Department of Women’s and Children’s Health, Division of Obstetrics and Gynecology, Karolinska Institutet, and Karolinska University Hospital, Stockholm, Sweden; ^3^ Celvia CC AS, Tartu, Estonia; ^4^ Tartu University Hospital Women’s Clinic, Tartu, Estonia; ^5^ Division of Obstetrics and Gynaecology, Department of Clinical Science, Intervention and Technology (CLINTEC), Karolinska Institutet, and Karolinska University Hospital, Stockholm, Sweden; ^6^ Institute of Genomics, University of Tartu, Tartu, Estonia; ^7^ Institute of Chemistry, University of Tartu, Tartu, Estonia; ^8^ Department of Hematology and Oncology, Institute of Clinical Medicine, University of Tartu, Tartu, Estonia

**Keywords:** endometriosis, hypoxia, stromal cell cultures, transcriptomics, peritoneal lesions, TGFBI

## Abstract

**Background:**

Endometriosis is characterized by the ectopic growth of endometrial-like cells, causing chronic pelvic pain, adhesions and impaired fertility in women of reproductive age. Usually, these lesions grow in the peritoneal cavity in a hypoxic environment. Hypoxia is known to affect gene expression and protein kinase (PK) activity. We aimed to explore the changes in the transcriptome and PK activity characteristic of eutopic and ectopic endometrium in endometriosis under hypoxia.

**Methods:**

Eutopic (EuESCs) and ectopic (EcESCs) endometrial stromal cells were exposed to hypoxia (1% O_2_) or normoxia (20% O_2_) for 48 hours. We assessed PK activity and examined transcriptome using mRNA-seq in cells cultured under hypoxic or normoxic conditions. Enzyme-linked immunosorbent assay, quantitative reverse transcription-PCR and immunohistochemistry were performed for the downstream analysis of Transforming Growth Factor Beta Induced (TGFBI) expression.

**Results:**

The kinase assay revealed a minor decrease in cAMP-dependent PK (PKAc) and Akt activity and a trend towards an increase in Rho-dependent PK (ROCK) activity in response to exposure to hypoxic conditions in EcESCs. A wider examination of the hypoxia-mediated changes in transcriptomes of cultured cells revealed that the genes related to aerobic glycolysis and cellular metabolism were upregulated in EuESCs exposed to hypoxia. In contrast, EcESCs had a single differentially expressed gene (*TGFBI*) upregulated under hypoxic conditions. This gene was also found to be overexpressed in EuESCs exposed to hypoxia vs normoxia, and in EcESCs vs EuESCs in normoxia. The level of secreted TGFBI in the spent culture media was accordingly high in the EcESC cultures and in the EuESC culture exposed to hypoxia. In the eutopic endometrial tissue biopsies, *TGFBI* mRNA and protein expression depended on the menstrual cycle phase, with higher levels observed in the proliferative phase. TGFBI staining showed the protein localized to the stroma and around the blood vessels. In the secretory phase, TGFBI protein expression was stronger in ectopic endometrium compared to paired eutopic endometrium.

**Conclusions:**

Within this study, we showed hypoxia-mediated transcriptome changes characteristic of EuESCs and EcESCs and identified TGFBI as a potential therapeutic target for endometriosis due to its role in fibrosis and angiogenesis.

## 1 Introduction

In endometriosis, endometrial-like cells grow and form lesions (ectopic endometrium, EcE) outside the uterus in a hypoxic microenvironment of the peritoneal cavity. Hypoxia is known to play an important role in endometriosis by regulating cell metabolism, growth, adhesion, and angiogenesis in developing endometriotic lesions (1, 2). The markers of hypoxia, adhesion, and invasion were found to be increased in the ectopic endometrial stromal cells (EcESCs) compared to the eutopic endometrial stromal cells (EuESCs) in women with or without endometriosis (3). Alterations in energy metabolism favoring aerobic glycolysis and inhibiting oxidative respiration were also observed in the EcESCs (3, 4), resembling the metabolic switch in the Warburg effect characteristic of cancer cells (5, 6).

Several metabolic markers, including hypoxia-inducible factor 1-alpha (HIF1A), pyruvate dehydrogenase kinase 1 (PDK1), and lactate dehydrogenase A (LDHA) were reported to be elevated at RNA and/or protein levels in EcE (7, 8), together with an increase in glucose uptake, lactate production, oxygen consumption, and cellular adenosine triphosphate (ATP) levels (7). Lactate, known to promote cell migration and angiogenesis in cancer (9), has been shown *in vitro* to regulate various processes like cell migration, invasion, and proliferation in EcESCs (10). The importance of hypoxia in the development of endometriosis was also supported by a study showing its potentiating effect on HIF-1α and integrins expression and adhesion capacity of endometrial cells important in the context of fibrosis (11). Moreover, transforming growth factor β (TGF-β) signaling activated under hypoxic conditions plays an important role in pro-fibrotic processes in the development of endometriosis (12).

Hypoxia has been demonstrated to stimulate angiogenesis in endometriotic lesions, with the adjacent peritoneum as a source for vessel branching (13). The elevated levels of pro-angiogenic factors like vascular endothelial growth factor-A (VEGF-A) (14), a downstream effector of TGF-β1, have been identified in endometriotic lesions (13, 15). Both VEGF-A receptors and TGF-β1 receptors represent distinct families of receptor protein kinases (PKs) that induce downstream signaling upon binding of the activating factors. *In vitro* studies have revealed that hypoxia affects the activity and/or localization of intracellular PKs (16–18). Constituting a family of over 530 members, PKs are crucial mediators in different cellular processes that can be related both to the normal functioning of various tissues as well as pathological conditions (19). For instance, we have previously shown that the activity of kinases like cAMP-dependent protein kinase (PKAc), Rho-associated protein kinase (ROCK), protein kinase B (Akt/PKB), and casein kinase 2 (CK2) is altered during decidualization *in vitro* (20). Interestingly, these same kinases have been implicated in the pathogenesis of endometriosis (21–25) and may contribute to the adaptation of cells to metabolic changes to support the growth of endometriotic lesions in a challenging environment like hypoxia.

In this study, we aimed to investigate how exposure to a hypoxic environment affects the activity of PKs and transcriptomic profile of the stromal cells (SCs) of both eutopic (EuE) and EcE in endometriosis.

## 2 Materials and methods

### 2.1 Patient selection and sample processing

The study was approved by the Research Ethics Committee of the University of Tartu, Estonia (approval No 333/T-6), and all the study participants have provided written informed consent. The tissue samples were collected from patients undergoing surgery at the Tartu University Hospital (Tartu, Estonia) and with confirmed endometriosis in accordance with the revised American Society for Reproductive Medicine classification system (26). Women undergoing laparoscopy due to infertility or pelvic pain, and confirmed endometriosis-free, were enrolled as controls (women without endometriosis). Peritoneal lesions were removed by laparoscopic surgery, and the collection of endometrial biopsy samples from women with and without endometriosis was done with an endometrial suction catheter (Pipelle, Laboratoire CCD). The enrolled women had not received any hormonal therapy for at least 3 months before the sample collection. **Table 1** provides detailed information about the patients enrolled in this study. The collected tissues were cut into two portions followed by endometriotic tissue histological evaluation and confirmation, or cryopreservation in Dulbecco’s Modified Eagle’s Medium (DMEM, Gibco, Thermo Fisher Scientific), with 30% fetal bovine serum (FBS, Biowest) and 7.5% dimethyl sulfoxide Hybri-Max (Sigma-Aldrich). The cryopreserved samples were stored in liquid nitrogen until further use.

**Table 1:** Patient characteristics in the study population.

	ESC culturing	IHC		qRT-PCR	
	Paired EuE and EcE (N = 5)	EuE (ES, N = 17)	Paired EuE & EcE (N = 7)	EuE (ES, N = 43)	EuE (Non-ES, N = 24)
Age (years, SD)	33 ± 4	32 ± 5.6	33 ± 4.8	32 ± 5.5	32 ± 5.6
BMI (kg/m^2^, SD)	23 ± 4	22 ± 3.8	22 ± 3.6	23 ± 3.1	23 ± 3.9
N of children, SD	1 ± 1	0 ± 0.83	0 ± 0.77	0 ± 0.85	1 ± 0.83
Infertility^a^ (N, %)	2 (40%)	4 (22%)	0	11 (25.6%)	1 (4.5%)
Smoking (N, %)	2 (40%)	2 (11%)	2 (33.3%)	2 (4.7%)	0
ES stage (N, %)	I – II (3, 60%) III – IV (2, 40%)	I – II (7, 41%) III – IV (10, 59%)	I – II (5, 71%) III – IV (2, 29%)	I – II (25, 58%) III – IV (18, 42%)	NA
MC phase (N)	S (5)	P (6), S (11)	P (1), S (6)	P (9), S (34)	P (5), S (19)

a Infertility at the moment of enrolment in the study. BMI, body mass index; EcE, ectopic endometrium; ES, women with endometriosis; EuE, eutopic endometrium; IHC, immunohistochemistry; MC, menstrual cycle; N, number; NA, not applicable; non-ES, women without endometriosis; qRT-PCR, quantitative RT-PCR; P, proliferative phase; S, secretory phase; SD, standard deviation.

### 2.2 Stromal cell isolation and primary cell culturing

Primary endometrial SCs were isolated from EuE, and EcE (peritoneal lesions) as previously described (3). EuESCs and EcESCs were cultured up to three passages in phenol red-free DMEM (Gibco) supplemented with 10% charcoal-stripped FBS (Gibco) with penicillin/streptomycin/amphotericin B (100 U/mL, 100 μg/mL, and 0.25 μg/mL, respectively) at 37°C under 20% O_2_ and 5% CO_2_. The cells were cryopreserved (1 vial from 1 confluent 10 cm Petri dish) and stored in liquid nitrogen until further use for functional assays.

### 2.3 Incubation of cells in normoxic or hypoxic conditions

The cells were thawed, seeded onto 6-well plates (working volume of 2 mL per well, approximately 170,000 cells per well) and cultured in phenol red-free DMEM with 10% charcoal-stripped FBS and penicillin/streptomycin/amphotericin B at 37°C under 20% O_2_ and 5% CO_2_ for 24 hours. Next, the medium was exchanged (working volume of 3 mL per well) and half of the replicates were cultured at 20% oxygen (normoxia) and the other half at 1% oxygen (hypoxia) for 48 hours. The hypoxic gas mixture (1% O_2_, 5% CO_2_, 94% N_2_) was supplied by the gas controller into the incubator of Cytation 5 multi-mode reader system equilibrated at 37°C (BioTek).

After incubation, the medium was removed (1 mL aliquots of spent medium were frozen and subsequently used for the enzyme-linked immunosorbent assay, ELISA) and the wells were rinsed with PBS supplemented with Ca^2+^ and Mg^2+^ (Corning). The cells were then lysed either at room temperature using RLT buffer (Qiagen; for RNA extraction) or on ice using the Triton X-containing HEPES buffer (Calbiochem, Ferak; for protein kinase assay).

### 2.4 Protein kinase assay

The fresh lysates prepared as previously described were used for protein kinase assay (20). For each lysate, total protein concentration was measured using Bradford assay (Thermo Scientific) according to the manufacturer’s instructions. Bradford assay was carried out in 96-well clear flat bottom plates (Nunc™ 269620) and absorbance at 590 nm was measured using a PHERAstar multi-mode reader (BMG Labtech). Within each experiment, the total protein concentration of all collected lysates was equalized by suitable dilution with kinase assay buffer as previously described (20).

The protein kinase assay was performed according to the previously published protocol using in-house synthesized photoluminescent probes that gain long-lifetime luminescence in complex with basophilic PKs (probe ARC-1139) or acidophilic PKs (probe ARC-1530) and the following displacing compounds: H89 (Biaffin) in case of PKAc; Y-27632 (Cayman Chemical) in case of ROCK; GSK690693 (Tocris) in case of Akt; and CX-4945 (Synkinase) in case of CK2 (20, 27, 28). The assay was carried out in 384-well round-bottom low volume plates (Corning 4514) and the time-delayed photoluminescence measurements were performed using PHERAstar multi-mode reader (BMG Labtech) with the following parameters: excitation 337 (300–360) nm, emission 675 (50) nm for ARC-1139 probe or 590 (50) nm for ARC-1530 probe, 100 flashes per well, integration start 60 μs, integration time 400 μs.

### 2.5 RNA extraction

The total RNA was isolated from the SC culture lysates using RNeasy mini kit (Qiagen) following the manufacturer’s instructions. RNA samples were treated with DNase to remove DNA content with Ambion^®^ DNA-free™ DNase Treatment and Removal Reagents (ThermoFisher Scientific). RNA concentrations were measured with a Qubit Flex Fluorometer (Invitrogen) following the protocol from the Qubit High Sensitivity RNA assay kit.

### 2.6 mRNA library generation and sequencing

The DNA libraries were constructed following the Smart-seq2 protocol described previously (29) with 10 ng of each RNA sample, then enzymatically fragmented and tagged using Nextera XT kit (Illumina Inc) and IDT^®^ for Illumina^®^ DNA Unique Dual Index barcodes. The purification of the final amplified cDNA libraries was performed using AMPure XP beads (1:1 ratio for the sample vs beads). The quantification of cDNA libraries was performed using the Qubit 1X HS DNA assay kit (Invitrogen), followed by the assessment of library quality using a high-sensitivity DNA chip (Agilent) on the 2100 Bioanalyzer system (Agilent). The pooled libraries were subjected to next-generation sequencing on the Illumina NovaSeq 6000 sequencing platform using a 2 x 150 bp read length (Novogene, UK).

### 2.7 mRNA-seq data analysis

The raw RNA-seq reads were subject to quality control assessment using the FastQC program (version 0.11.8) (30). Next, a comprehensive report based on the FASTQC results was generated using the MultiQC program (31) to evaluate the sequencing data quality. The fastp program (32) was then utilized with default parameters to remove adaptor sequences and perform read trimming. The resulting trimmed reads were aligned to the human reference genome GRCh38 using STAR 2.7.5a (33), accounting for paired-end reads. Gene counts were derived from the aligned reads utilizing the featureCounts program (34) with default parameters, also considering paired-end reads. On average, mRNA-seq generated 5.2 million reads per sample, ranging from 3.5 million to 7.2 million reads.

Principal component analysis (PCA) was performed using the plotPCA function available in the DESeq2 R package (35). Prior to performing PCA, the raw count data underwent variance stabilizing transformation (VST) to alleviate potential biases and improve clustering accuracy. For differential gene expression analysis, the DESeq2 R package (35) was utilized. We applied gene filtering to include only those genes with at least ten counts across all samples in at least one of the experimental groups. Next, pairwise differential gene expression analysis was conducted between experimental groups indicated in **Table 2**. The resulting p-values underwent adjustment using Benjamini and Hochberg’s (BH) method to control the false discovery rate (FDR). Genes exhibiting an absolute log_2_ fold change |log_2_FC| > 0.5 and an FDR-adjusted P-value (P_adj_) < 0.05 were considered differentially expressed.

**Table 2:** The numbers of statistically significant upregulated and downregulated DEGs between comparison groups.

Comparison groups			Statistically significant DEGs, # (P_adj_ < 0.05, log_2_FC ≤ –0.50 or ≥ 0.50)			
#	Group 1	Group 2	Total, #	Up, #	Down, #	*TGFBI*, log_2_FC
1	EcESCs_H	EcESCs_N	1	1	0	2.0
2	EuESCs_H	EuESCs_N	116	116	0	2.2
3	EcESCs_H	EuESCs_H	20	5	15	1.8
4	EcESCs_N	EuESCs_N	163	142	21	1.9
5	EcESCs_H	EuESCs_N	569	194	375	3.9

EuESCs, eutopic endometrial stromal cells; EcESCs, ectopic endometrial stromal cells; H, exposure to hypoxia; N, exposure to normoxia; DEGs, differentially expressed genes; log_2_FC, log_2_ fold change; #, number; Up and Down, upregulated or downregulated genes.

Pathway analysis was performed using the g:Profiler web tool (36). Specifically, the g:GOSt function within g:Profiler facilitated gene-set enrichment analysis, with significance determined using the tailored significance threshold g:SCS. KEGG and GO: BP plots were made using the online tool ShinyGO 0.80 (37–39). Venn diagrams were made using the jvenn online tool (40). Only the genes with nomenclature names were included in the list used for creating the Venn diagram.

### 2.8 Enzyme-linked immunosorbent assay

The amount of secreted TGFBI protein was quantified in spent cell culture media using the EHTGFBI (BIGH3) human ELISA kit (Invitrogen) according to the manufacturer’s instructions. Absorbance at 450 nm was measured using a Cytation 5 multi-mode reader (BioTek).

### 2.9 qRT-PCR

cDNAs were synthesized by RevertAid First Strand cDNA Synthesis Kit (Thermo Scientific). The qRT-PCR analysis for transforming growth factor beta induced (*TGFBI*) expression was performed using 5x HOT FIREPol EvaGreen qPCR Mix Plus (ROX) (Solis BioDyne) with the primers (F: 5´-TAACGGCCAGTACACGCTT-3´; R: 5´-TGTTCAGCAGGTCTCTCAGG-3´). Gene expression data was normalized to the *SDHA* housekeeping gene (F: 5’-TGGGAACAAGAGGGCATCTG-3’; R: 5’-CCACCACTGCATCAAATTCATG-3’) as described previously (41).

### 2.10 Immunohistochemistry

To localize TGFBI protein in EuE and EcE, IHC analysis of tissue samples was performed. The tissue specimens were fixed in 10% neutral buffered formalin, dehydrated and embedded in paraffin at TUH’s Pathology Service. The prepared 2.5 µm tissue sections were deparaffinized in xylene followed by rehydration with a series of washes (2x, each for 5 min) in ethanol (96%, 90%, 80%, 70%, 60%, 50%) and MQ water. Next, tissue sections were incubated for 20 min at 98°C in 10 mM sodium-citrate buffer (pH = 6.0) for antigen retrieval and 5% normal goat serum in 1% BSA/PBS was used for 60 min to block nonspecific binding. The following steps were performed according to the Master Polymer Plus Detection System (Peroxidase) (Incl. 3,3’-Diaminobenzidine (DAB) Chromogen) Kit (Master Diagnostica, Spain) guidelines. All the washes were performed with 1x TBS (Fisher Bioreagents, USA), 3 times, each for 5 min. To identify TGFBI protein, tissue sections were incubated with rabbit anti-TGFBI antibody (Invitrogen, JM24-53, catalogue N MA5-32736, dilution 1:100) overnight at 4°C, and as negative controls, the tissue sections were incubated with 1% BSA/PBS. For nuclear counterstain, Mayer hematoxylin solution 1:4 was used. Lastly, the tissue sections were dehydrated in the opposite order as for the rehydration procedure, by incubating in ethanol and xylene. A mounting medium (Leica CV mount, Leica Biosystems, USA) was applied to the tissue sections and covered by coverslips. The tissue sections were then scanned with a Leica SCN400 slide scanner (Leica Biosystems, USA) using a 20x objective.

Semi-quantitative analysis of scanned sections was performed using the ImageJ Fiji package (v. 1.54k) (42). Briefly, DAB signal intensity was measured in the endometrial stroma, excluding luminal and glandular epithelium. The relative DAB intensity was calculated using the formula: f = 255 - i, where “f” is relative DAB intensity and “i” is mean DAB intensity obtained from the software ranging from 0 (deep brown, highest expression) to 255 (total white) (43).

### 2.11 Statistical analysis

PK assay: The initial normalization of signals was carried out according to the kinase activity profile observed in EuESCs under normoxic conditions (set to 100% for each PK of interest). Due to a large spread of data points observed for EcESCs, the second round of normalization was then carried out where EcESCs were normalized separately from EuESCs (cells treated under normoxia set to 100% for each PK of interest). The pairwise comparisons between the PK activity profiles in different oxygenation conditions or eutopic vs ectopic cell lysates (N = 5) were carried out using the unpaired two-tailed t-test with Welch’s correction and the significance of comparisons is indicated as follows: ** corresponds to P ≤ 0.01, * corresponds to P ≤ 0.05.

ELISA assay: For each sample of spent medium, the calculated TGFBI concentration was normalized to the total protein content measured for the corresponding cell lysate (*i.e*., homogenate of cells growing in the well from which the medium sample was initially collected) as described in Section 2.4. An additional normalization was carried out where for samples obtained from the same patient, the relative TGFBI amount was normalized according to the signal measured for the EuESCs under normoxia (set to 100%) or for the EuESCs or EcESCs incubated in normoxia (= 100%) separately. The pairwise comparisons between the oxygenation conditions or eutopic vs ectopic cells (N = 5) were carried out using the unpaired two-tailed t-test with Welch’s correction and the significance of comparisons is indicated as follows: *** corresponds to P ≤ 0.001, ** corresponds to P ≤ 0.01.

qRT-PCR: The comparison between *TGFBI* mRNA expression levels (ΔCt values) in proliferative vs secretory phase endometrium from women with (N = 45) and without (N = 24) endometriosis was performed using the unpaired two-tailed t-test with Welch’s correction and the significance of comparisons is indicated as follows: **** corresponds to P < 0.0001.

IHC: Statistical significance was assessed using the Wilcoxon Mann-Whitney test, with *corresponding to P ≤ 0.05.

For statistical analysis of data from ELISA, PK assay, qRT-PCR, and IHC, GraphPad Prism 6 (San Diego) was used as reported previously (20).

## 3 Results

### 3.1 The activity of PKAc and Akt kinase families is reduced in the hypoxia-treated EcESCs but not in the EuESCs

We started our study by examining the activity profiles of PKs that have been previously shown to contribute to the decidualization of normal endometrium – with PKA, ROCK and Akt/PKB featuring increased activity and CK2 featuring decreased activity in the ESCs decidualized *in vitro* or *in situ*. Our current goal was to establish whether the activity of these kinases can be altered depending on the origin of cells (eutopic vs ectopic) or short-term changes in oxygenation conditions (20% vs 1% oxygen). The activities of four PK families – PKAc, ROCK, Akt, and CK2 – were measured in fresh lysates prepared directly after incubation of EuESCs and EcESCs under different oxygenation conditions. To compensate for the differences in number of cells following the 48-h incubation, the lysates obtained from the same patients’ samples were diluted to the same total protein content. For each individual patient, the relative PK activity was normalized based on the PK activity established for EuESCs in normoxia, which was set to 100%.

**Figure 1:**
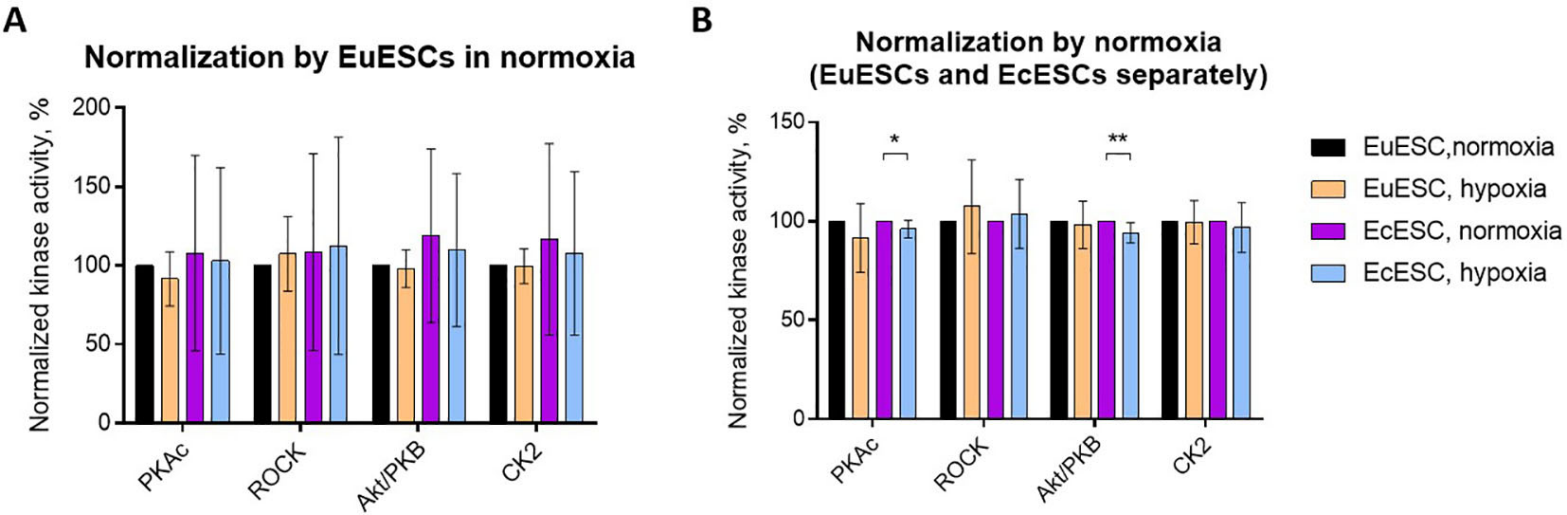
Protein kinase activity profiles in lysates of EuESCs or EcESCs incubated in different oxygenation conditions. The normalization was done by EuESCs in normoxia **(A)** or by normoxia in EuESCs and EcESCs separately set to 100% **(B)**. The PKs of interest are listed below the graph and the types of cells and incubation conditions are on the right. EuESCs, eutopic endometrial stromal cells; EcESCs, ectopic endometrial stromal cells; PKAc, cAMP-dependent PK; ROCK, Rho-dependent PK; Akt/PKB, protein kinase B; CK2, casein kinase 2. Each column shows the mean ± standard deviation for samples obtained from 5 different patients. Asterisks indicate pairwise comparisons (t-test with Welch’s correction): **P ≤ 0.01, *P ≤ 0.05; only statistically significant comparisons are shown.

The pooled results (N = 5) are summarized in **Figure 1A**; it was evident that the comparison of EuESCs and EcESCs was masked by the large interpatient variation. Hence, we focused on the comparisons of oxygenation conditions and carried out separate normalization for EuESCs and EcESCs (i.e., data for EcESCs incubated in normoxia was also set to 100% for each individual PK; **Figure 1B**). The obtained PK activity profiles revealed subtle yet statistically significant decrease in PKAc and Akt activity for EcESCs incubated in hypoxia vs normoxia (P < 0.05 and P ≤ 0.01, respectively). The trends were similar in the case of EuESCs incubated in hypoxia vs normoxia, yet statistical significance could not be achieved. No clear trends were observed in the activity of CK2, whereas in the case of ROCK, a slight elevation of kinase activity under hypoxic conditions was evident, which was however not statistically significant.

As the PK assay revealed only minor changes in the examined PK pathways which could not be explained solely by the effect of hypoxia, we decided to assess a wider picture of putative differences between the EuESC and EcESC signaling pathways under different oxygenation conditions by exploring the transcriptome.

### 3.2 Hypoxia alters the transcriptome of endometrial SCs with distinct differences between EuESCs and EcESCs

To explore how hypoxia affects the gene expression of SCs isolated from paired EuE and EcE of women with endometriosis, we sequenced the entire transcriptome of the cell culture samples exposed to both normoxia and hypoxia (EuESCs, N = 5 and EcESCs, N = 5, in total 20 samples). By applying the gene filtering procedure as mentioned in the methods section, 17,887 genes were retained for subsequent analyses. The principal components analysis (PCA, **Supplementary Figure 1**) showed no specific grouping of samples based on the correlation between the data points representing each sample in four study groups. The Pearson correlation analysis revealed high intra-group variability in EuESCs exposed to hypoxia and EcESCs exposed to normoxia (**Supplementary Figure 2**). However, there was a strong similarity at the transcriptome level between all the samples in the study groups based on the correlation coefficient values ranging from 0.85 to 1.

**Figure 2:**
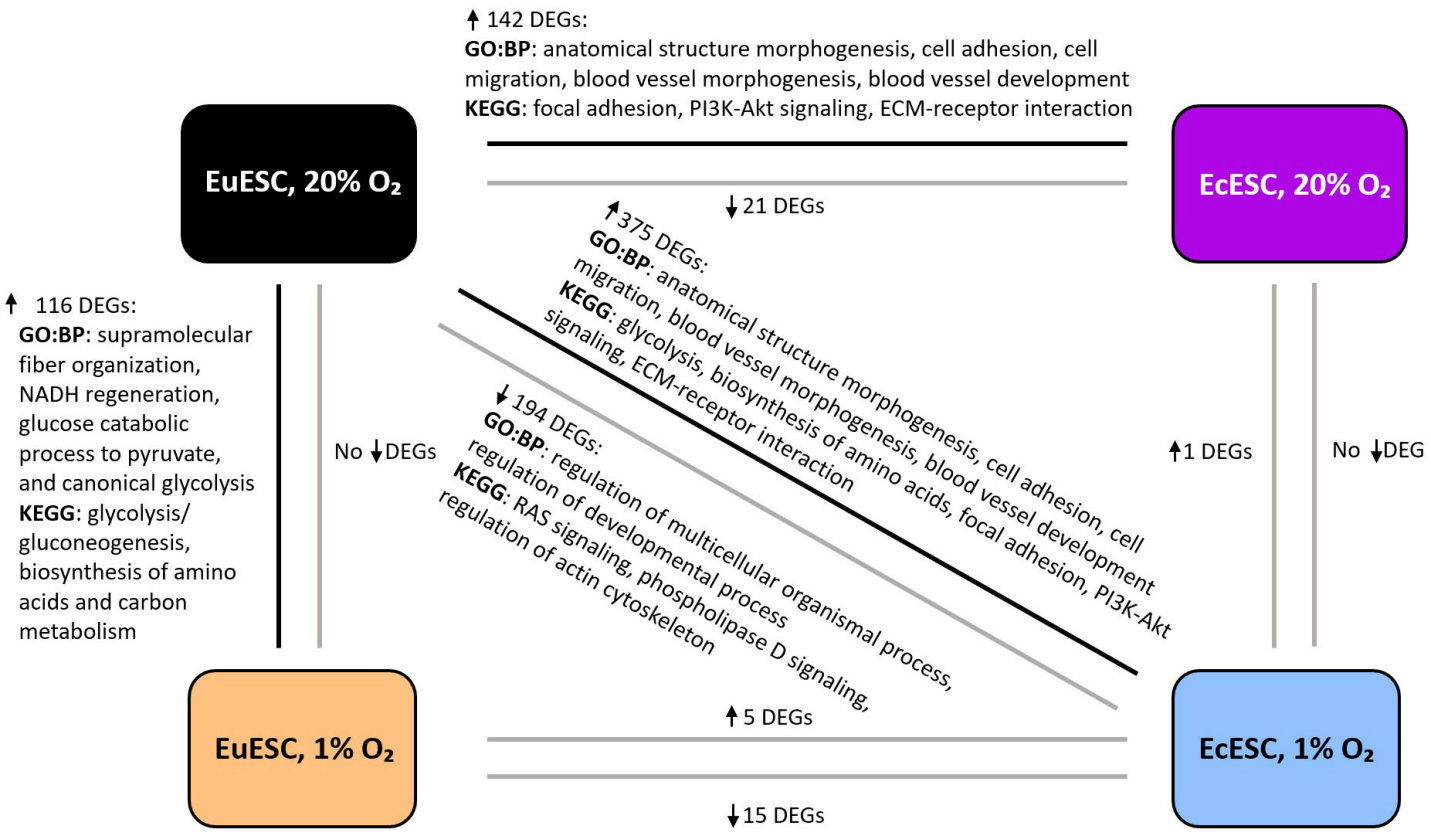
The representative biological processes (GO: BP) and enriched pathways (KEGG) in the comparison groups. The analysis is based on statistically significant DEGs, P_adj_ < 0.05). DEGs, differentially expressed genes; EuESCs, eutopic endometrial stromal cells; EcESCs, ectopic endometrial stromal cells. The arrows next to the DEGs indicate up- or down-regulation.

The comparisons of EuESCs and EcESCs exposed to hypoxia and/or normoxia yielded different numbers of differentially expressed genes (DEGs) (the comparison groups and numbers of DEGs are shown in **Table 2**, statistically significant DEGs presented in **Supplementary Table 1**). The gene ontology and pathway enrichment analyses of DEGs in comparison groups identified statistically significantly enriched biological processes and pathways presented in **Figure 2** and **Supplementary Table 2**.

In EuESCs, we identified 116 DEGs upregulated under hypoxic conditions (log_2_FC range from 0.5 to 2.9). Gene set enrichment analysis showed glycolysis/gluconeogenesis, biosynthesis of amino acids and carbon metabolism pathways among the top enriched pathways, which correlated with HIF-1 signaling pathway via shared DEGs (**Supplementary Figure 3A**). Among the top biological processes, the analysis revealed supramolecular fiber organization, NADH regeneration, glucose catabolic process to pyruvate, and canonical glycolysis (**Supplementary Figure 3B**). The expression profiles of EcESCs exposed to hypoxia vs normoxia were similar and the analysis identified a single statistically significant DEG, *TGFBI* (log_2_FC 2.0).

In the comparison group of both EcESCs and EuESCs exposed to hypoxia, we identified 20 DEGs, of which 15 were downregulated (log_2_FC range from –8.8 to –0.8) and 5 upregulated (log_2_FC range from 0.7 to 3.4). The comparison of EcESCs and EuESCs both exposed to normoxia revealed 163 DEGs, 142 of which were upregulated (log_2_FC range from 0.5 to 6.9) and 21 downregulated (log_2_FC range from –0.5 to –2.3) in ectopic samples. Among the top enriched KEGG pathways in the upregulated DEG group, we identified focal adhesion, PI3K-Akt signaling pathway, and extracellular matrix (ECM)-receptor interaction (**Supplementary Figure 3C**). The anatomical structure morphogenesis, cell adhesion, cell migration, blood vessel morphogenesis, and blood vessel development were among the top enriched biological processes (**Supplementary Figure 3D**).

Given that EcE resides *in vivo* at more hypoxic conditions than the EuE, we hypothesized that in the case of EcESCs, incubation in a hypoxic environment closely resembles the physiological conditions, while in the case of EuESCs, hypoxia induces reductive stress. We therefore proceeded with a comparison of cell transcriptomes mirroring the native-like states, i.e., EcESCs exposed to hypoxia compared to EuESCs exposed to normoxia. We found 569 DEGs, with 375 upregulated genes (log_2_FC range from 0.5 to 7.7) and 194 downregulated genes (log_2_FC range from –0.5 to –8.7). The analysis of upregulated genes showed sugars and amino acids metabolism pathways, glycolysis, ECM-receptor interaction, focal adhesion, and PI3K-Akt signaling pathway and wound healing, blood vessel development, actin cytoskeleton development processes among the top enriched pathways and biological processes (**Supplementary Figures 3E, F**). In the downregulated DEGs group, RAS signaling and regulation of actin cytoskeleton were among the top KEGG pathways, and the regulation of cell differentiation and regulation developmental process were among the top biological processes (**Supplementary Figures 3G, H**).

**Figure 3:**
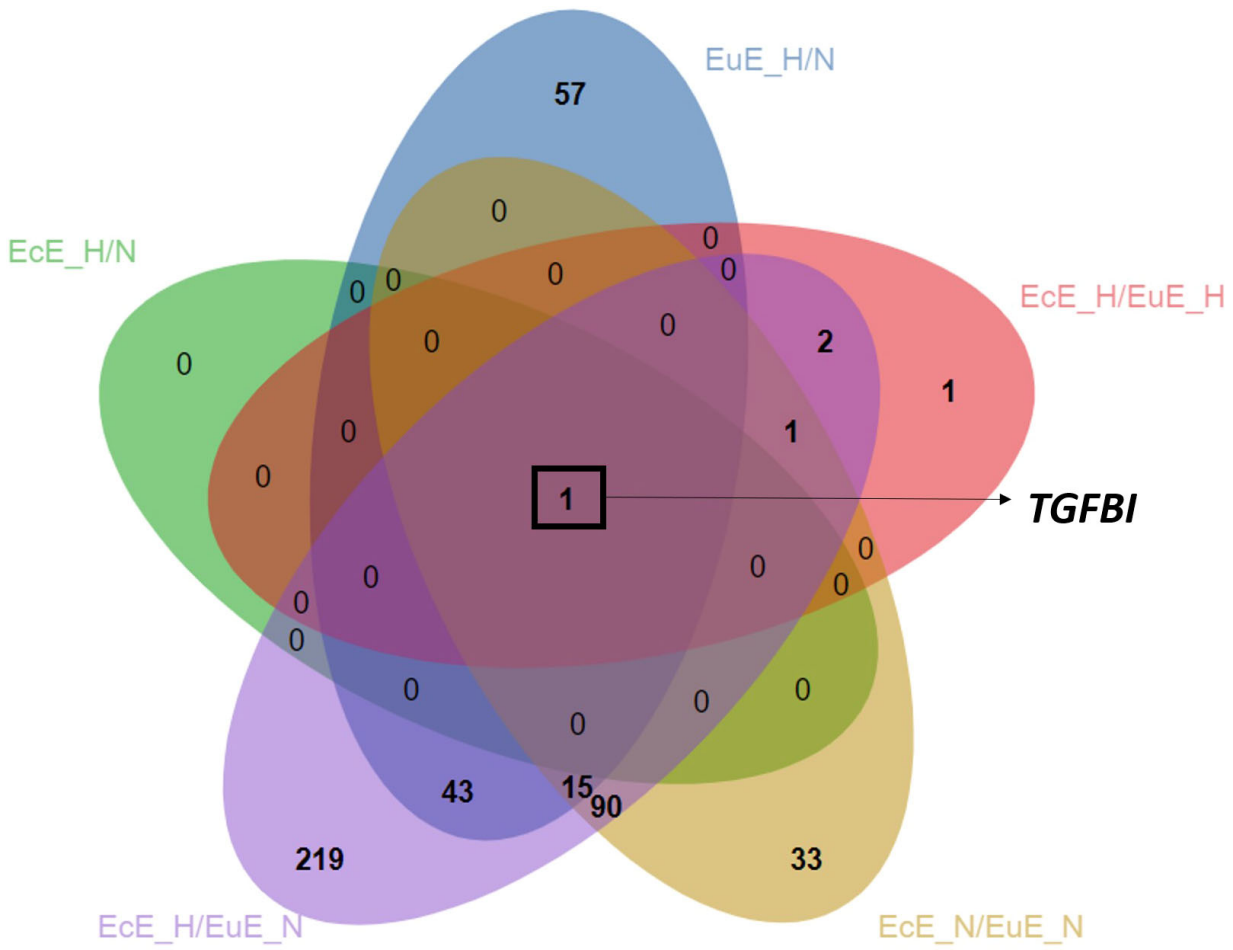
Venn diagram depicting overlapped upregulated DEGs between comparison groups. The analysis is based on statistically significant DEGs, P_adj_ < 0.05). EcE_H/N, EcESCs exposed to hypoxia vs normoxia; EuE_H/N, EuESCs exposed to hypoxia vs normoxia; EcE_H/EuE_H, EcESCs vs EuESCs both exposed to hypoxia; EcE_N/EuE_N, EcESCs vs EuESCs both exposed to normoxia; EcE_H/EuE_N, EcESCs exposed to hypoxia vs EuESCs exposed to normoxia; EuESCs, eutopic endometrial stromal cells; EcESCs, ectopic endometrial stromal cells; DEGs, differentially expressed genes. The novel transcripts corresponding to genes with no nomenclature names were excluded from the list used for creating the Venn diagram, resulting in a smaller number of DEGs in the following groups: EcESCs vs EuESCs exposed to normoxia, and EcESCs exposed to hypoxia vs EuESCs exposed to normoxia.

The Venn diagram showed that the focal adhesion and the regulation of actin cytoskeleton KEGG pathways were shared among the three groups: EuESCs exposed to hypoxia vs normoxia, EcESCs vs EuESCs exposed to normoxia, and EcESCs exposed to hypoxia vs EuESCs exposed to normoxia (**Supplementary Figure 4**). The analysis of common DEGs in the 5 comparison groups indicated that *TGFBI* was upregulated in all the comparisons (**Table 2**; **Figure 3**).

### 3.3 Secretion of TGFBI mirrors transcriptome changes in ESCs

According to previous studies, TGFBI is a secreted protein (44, 45). To establish whether the trends observed on the level of transcriptome are also valid at the protein level, we quantified the amount of secreted TGFBI in the spent cell culture media using ELISA. To compensate for the differences in the number of TGFBI-secreting cells following the 48-h incubation in different oxygenation conditions, the ELISA data was normalized to the total protein content in lysates of cells from which the media aliquots were collected. Furthermore, to compensate for the interpatient variation in TGFBI secretion, for each patient, the relative TGFBI protein content was additionally normalized according to that established for the EuESCs in normoxia (set to 100%).

**Figure 4:**
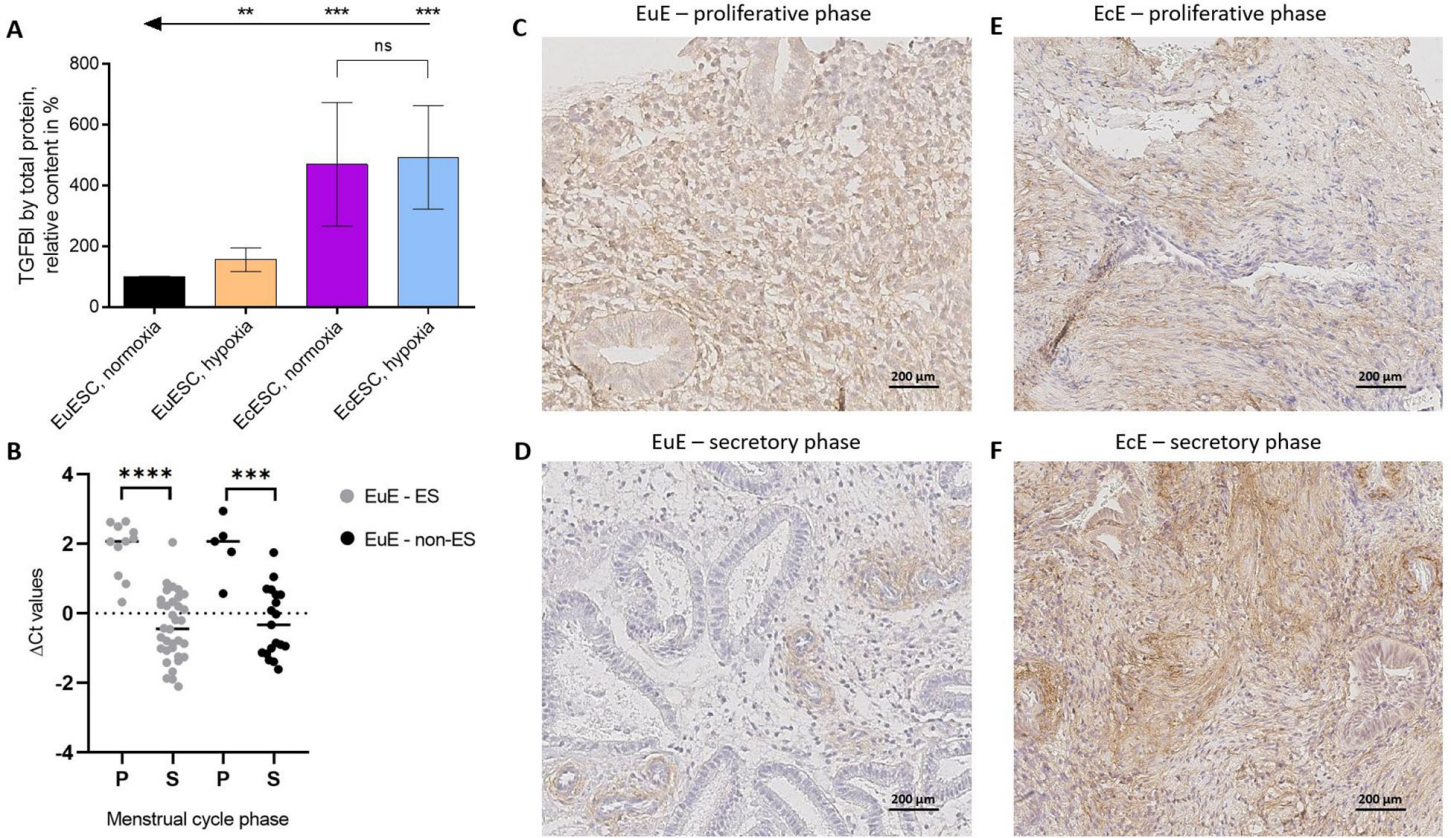
The confirmation of TGFBI’s importance in the context of endometriosis on the levels of protein and mRNA. **(A)**. Secreted TGFBI protein levels in spent cell culture media relative to the total protein content in the corresponding cell lysates. For each patient, the data was normalized to the relative TGFBI content measured for the EuESCs incubated in normoxia (= 100%). Each column shows the mean ± standard deviation for samples obtained from 5 different patients. Arrows and asterisks indicate pairwise comparisons (t-test with Welch’s correction): ***P ≤ 0.001, **P ≤ 0.01. **(B)**. *TGFBI* mRNA expression in eutopic endometrial tissues from women with (light grey) or without (black) endometriosis (N = 45 and N = 24, respectively) in proliferative (P) and secretory (S) phases of menstrual cycle. ΔCt values correspond to the relative expression level of *TGFBI*, the thick line is the median. An unpaired two-tailed t-test with Welch’s correction was applied, ****P value < 0.0001, ***P value < 0.001. **(C–F)**. TGFBI protein localization in EuE and EcE from women with endometriosis at the proliferative **(C, E)** and secretory **(D, F)** phases of the menstrual cycle. Scale bar 200 μm. EcESCs, ectopic endometrial stromal cells; EuESCs, eutopic endometrial stromal cells; EuE, eutopic endometrium; EcE, ectopic endometrium; ES, endometriosis; non-ES, non-endometriosis; ns, not significant.

The results (N = 5 in each group) are summarized in **Figure 4A** and **Supplementary Figure 5**. Expectedly, relative TGFBI secretion was elevated in the medium collected from EuESCs incubated under hypoxic vs normoxic conditions (P < 0.01), and in the medium collected from EcESCs in both conditions relative to EuESCs (P < 0.001). In the case of EcESCs, no significant difference in TGFBI secretion was observed dependent on the oxygenation conditions, yet the trend was similar to that observed in transcriptomics with higher *TGFBI* levels in EcESCs exposed to hypoxia. Furthermore, in the case of samples collected from EcESCs in hypoxia, the TGFBI levels were closer to the upper limit of the calibration curve, thus potentially complicating the comparison.

### 3.4 The mRNA expression pattern of *TGFBI* in EuE from women with and without endometriosis is dependent on the menstrual cycle phase

As the isolated SCs represent a simplified model system, we proceeded to investigate the *TGFBI* in the context of endometriosis in a physiologically more relevant system represented by the full tissue samples. The ESCs used in our experiments were cultured in the absence of hormones to achieve a sufficiently numerous and homogenous cell population. As a result, the cells likely lost the characteristics of the menstrual cycle phase, at which the patient samples were initially collected (46). Therefore, we decided to investigate whether *TGFBI* mRNA expression varies depending on the menstrual cycle phase. For that, we determined *TGFBI* mRNA level in 45 EuE from women with endometriosis (11 in the proliferative phase and 34 in the secretory phase), and 24 EuE from women without endometriosis (controls, 5 in the proliferative phase and 19 in secretory phase) using qRT-PCR.

We found *TGFBI* mRNA expression in EuE from both women with endometriosis and controls to be menstrual cycle phase-dependent, with a higher expression level in the proliferative phase compared to the secretory phase (FC = 4.5, P < 0.0001 and FC = 4.5, P < 0.001, respectively, **Figure 4B**). Having confirmed that *TGFBI* mRNA levels are dependent on the menstrual cycle phase, we proceeded to the exploration of TGFBI protein expression and localization in tissue samples from women with endometriosis obtained at proliferative or secretory phases.

### 3.5 TGFBI protein is expressed in the stroma and around the vessels of EuE and EcE

To determine which cell types express TGFBI protein in endometrium and lesions, IHC was performed on paired samples of EuE and EcE from seven women with endometriosis, and additionally 17 EuE from women with endometriosis (samples collected during the proliferative or secretory phases of the menstrual cycle).

We observed a moderate and diffuse TGFBI protein expression in the stroma of paired EuE during the proliferative phase of the menstrual cycle (**Figure 4C**; **Supplementary Figure 6**). In the secretory phase, TGFBI protein staining in stroma was sparse and focal, localized around the vessels (**Figure 4D**; **Supplementary Figure 6**). The tissue sections of paired EcE contained fibrotic areas, and TGFBI protein expression was diffuse in stroma and around the vessels (**Figures 4E, F**; **Supplementary Figure 6**). In the secretory phase, TGFBI protein expression was stronger in EcE compared to EuE, which is similar to our observations in the SC culture experiments. Additionally, we analyzed TGFBI protein signal intensity in 17 EuE from women with endometriosis (6 in proliferative phase and 11 in secretory phase). TGFBI protein expression was significantly higher in the proliferative phase compared to the secretory phase (P < 0.05, **Supplementary Figures 7**-**9**). Due to the fibrotic nature of EcE, we did not compare TGFBI signal intensity between 6 paired EuE and EcE samples in the secretory phase to avoid potential artifacts.

## 4 Discussion

**Figure 5:**
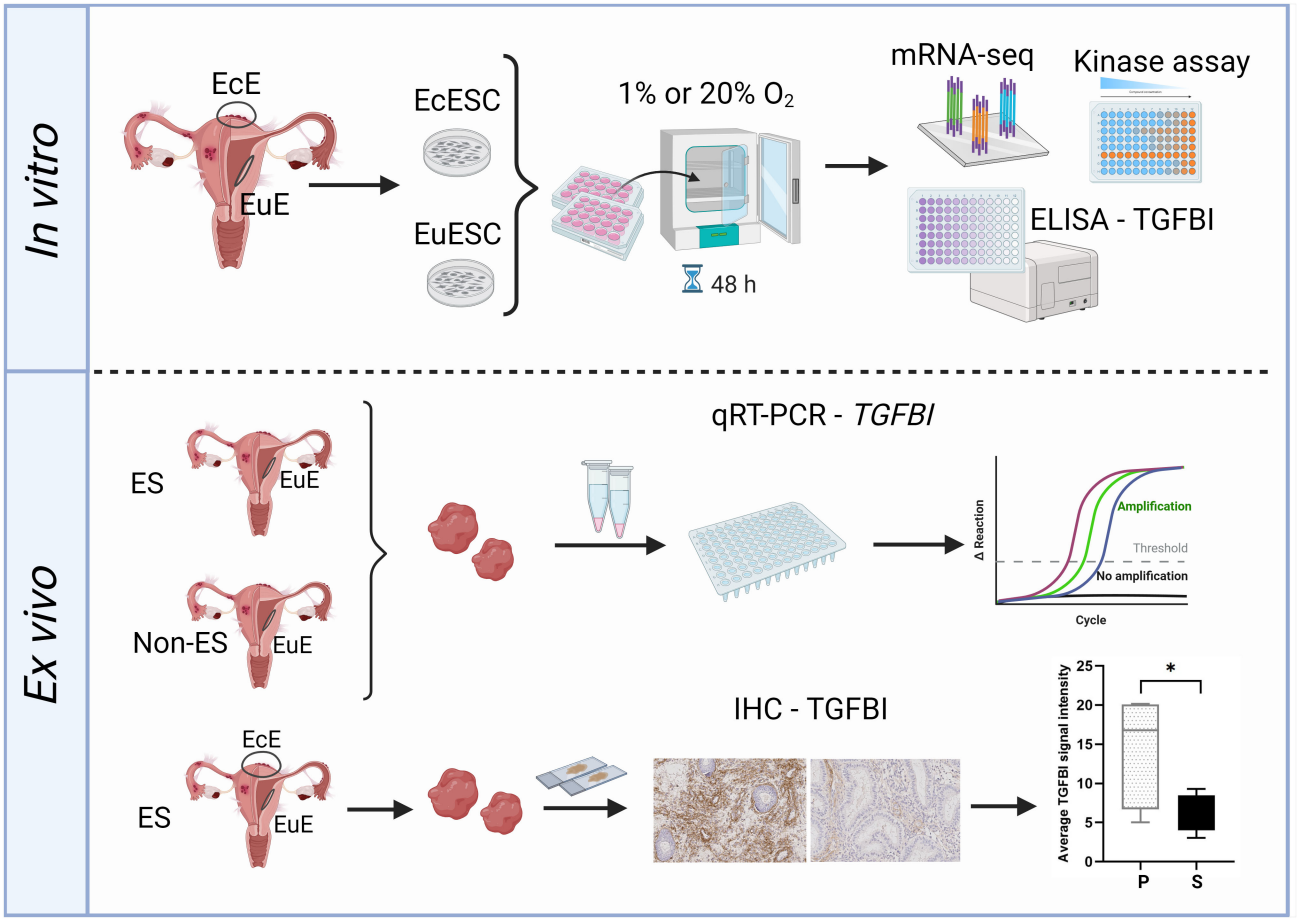
The study workflow overview. Upper panel: ectopic endometrial stromal cells (EcESCs) and eutopic endometrial stromal cells (EuESC) were isolated from ectopic endometrium (EcE) and eutopic endometrium (EuE), respectively. The primary cell cultures were exposed to hypoxic (1% O_2_) or normoxic (20% O_2_) conditions for 48 hours followed by examination of gene expression (mRNA-seq), assessment of kinase activity (kinase assay) and detection of TGFBI level in culture media (ELISA assay). Lower panel: the samples of EuE from women with and without endometriosis were subjected for qRT-PCR to assess the level of expression of *TGFBI*. EuE and EcE from women with and without endometriosis were used for immunohistochemistry (IHC) for TGFBI protein localization and quantification of TGFBI signal intensity. The Figure was created with BioRender.com (representative IHC images were obtained in this study). EcE, ectopic endometrium; EuE, eutopic endometrium; EcESCs, ectopic endometrial stromal cells; EuESCs, eutopic endometrial stromal cells; ES, endometriosis; Non-ES, non-endometriosis; IHC, immunohistochemistry.

Hypoxic microenvironment was proposed to drive the pathogenesis of endometriotic lesions, promoting processes like angiogenesis, cell survival and metabolic changes (2). Our study investigated the effect of short-term (48 hours) hypoxic treatment on EuESCs and EcESCs by assessing the activity of a targeted set of physiologically relevant kinases and performing a global transcriptome profiling. All methods converged to TGFBI as the important player from the point of both endometriosis and hypoxia (**Figure 5**).

In EuESCs, mRNA-seq analysis revealed hypoxia-mediated activation of biological processes related to aerobic glycolysis, NADH regeneration, and pathways, such as glycolysis/gluconeogenesis, biosynthesis of amino acids, and carbon metabolism (**Figure 2**). These results were in concordance with previous reports on the increased expression of hypoxia-induced markers of aerobic glycolysis like LDHA and PDK1 in EuESCs (7, 8). The expression of these markers was also associated with decreased apoptosis, which has been proposed to give an advantage to EuESCs during the establishment and development of endometriotic lesions. The mitochondrial oxygen consumption rate and the levels of metabolites from biosynthesis of amino acids pathway in EcE and EuE has have been shown to be decreased in EuE and EcE from endometriosis model compared to control endometrium, as well as the levels of metabolites from biosynthesis of amino acids pathway in an endometriosis model (47). In our study, EcESC cultures exposed to hypoxia vs normoxia had a single DEG, *TGFBI*, upregulated in cells exposed to hypoxia (**Table 2**). The low number of DEGs in EcESCs, regardless of oxygen level in culture conditions, might refer to a possibility that EcESCs had already been epigenetically adjusted for low oxygen concentration in lesions - and such reprogramming persisted during cultivation *in vitro*. When we compared both EcESCs exposed to hypoxia or normoxia to EuESCs exposed to normoxia, we identified enriched processes like anatomical structure morphogenesis, cell adhesion, cell migration, blood vessel morphogenesis, and blood vessel development (**Figure 2**; **Supplementary Figures 3D, F**). Several studies have demonstrated that exposure of EuESCs and EcESCs to hypoxia alters expression of genes like *DUSP2*, *IL-8*, *COUP-TFII*, *ANG*, and leptin, involved in the activation of tube formation and angiogenesis (48–50).

*TGFBI* was a common gene overexpressed in EcESCs under hypoxia or normoxia and EuESCs exposed to hypoxia (**Table 2**; **Figure 3**). The ELISA assay with spent cell culture media confirmed the increased secretion of TGFBI protein in hypoxic vs normoxic conditions and in EcESCs vs EuESCs (**Figure 4A**). Recent studies have demonstrated the increased levels of TGFBI protein in peritoneal fluid and serum of women with endometriosis compared to controls (44, 45). However, its role in endometriosis pathogenesis was not yet explored. The gene ontology (molecular function) analysis of our mRNA-seq data has shown that TGFBI is involved in cell adhesion molecule binding, extracellular matrix structural constituent, and integrin binding (**Supplementary Table 2**). The biological processes, in which TGFBI is involved, included blood vessel development, morphogenesis, angiogenesis, cell adhesion, cell differentiation, and cellular developmental process. However, the reports on the role of TGFBI in angiogenesis are controversial. It was reported to inhibit adipose angiogenesis (51), while promote angiogenesis in lungs (52). Moreover, TGFBI was found to have pro-fibrotic effect in lung tissues. Its induction by TGF-β1 in lung fibroblasts led to overexpression of collagen I and α-SMA via TGFBI/GPSM2/Snail axis, whereas the knockdown of TGFBI had a reverse effect on the pro-fibrotic process (53). TGF-β1 has been shown to activate epithelial-to-mesenchymal and fibroblast-to-myofibroblast transition, leading to increased production of collagen *in vitro* and fibrogenesis *in vivo* in endometriosis (54, 55). TGFBI might mediate fibrotic process in endometriosis, therefore targeting TGFBI should be investigated as a potential strategy to reduce fibrosis in endometriosis models.

Furthermore, the putative effect of TGF-β1/TGFBI axis on the intracellular pathways was evident from the PK assay results (**Figures 1B, C**). Namely, according to the literature, hypoxic environment promotes activation of PKAc pathways, while its effect on Akt/PKB activity depends on the model system explored yet is also predominantly activating (16, 56–60). By contrast, we observed decrease in PKAc and Akt activity in EcESCs treated under hypoxic vs normoxic conditions, and the same trends were evident in EuESCs. This observation is rather in line with the previously reported effects of TGFBI, which suppresses both Akt/PKB and cAMP-dependent signaling pathways (61–63). In addition, the observed trend towards the increase in ROCK activity in EuESCs and EcESCs under hypoxic conditions can be explained by TGFβ-mediated activation of RhoA/ROCK signaling axis associated with fibrosis in the context of other tissues (64, 65). In fact, according to literature, TGFBI has been proposed to activate the focal adhesion kinase in some of the explored models (66, 67) – and focal adhesion kinase is also known to activate ROCK signaling in fibroblasts (68).

Apart from TGFBI, another downstream protein of TGF-β1 represented by the TAGLN (69, 70) can also affect the protein kinase pathways. In a different physiological context, TAGLN has been proposed to activate ROCK signaling in ovarian cancer (71) and downregulate Akt signaling in smooth muscles (72). Importantly, in literature, TAGLN has also been investigated in the context of endometriosis, showing increased gene expression in endometriotic lesions (73) and being upregulated by the pathogenic *Fusobacterium* in endometrium of endometriosis patients (74). According to the transcriptomic data in our study, TAGLN and its homolog TAGLN2 were overexpressed in EcESCs in hypoxia vs EuESCs in normoxia and EuESCs exposed to hypoxia vs normoxia (**Supplementary Table 1**). It is thus extremely important to explore in future studies whether the TGF-β1/TGFBI and TGF-β1/TAGLN signaling axes mediate and consolidate the hypoxia-driven and the inflammation-driven aspects of the endometriosis pathology.

While the experiments with isolated cultured SCs in our study consistently indicated the importance of hypoxia in EcESCs and the role of TGFBI in EcESC functioning, such *in vitro* model is simplified and does not account for the cell population heterogeneity in EuE or EcE, or for changes in cell phenotypes associated with progression through the menstrual cycle. Thus, we decided to explore TGFBI expression in a more physiologically relevant system represented by the EuE and/or EcE from women with endometriosis.

*TGFBI* mRNA expression in the bulk tissues of EuE from women with endometriosis and controls was higher in the proliferative phase of menstrual cycle compared to the samples from the secretory phase (**Figure 4B**). TGFBI immunostaining showed the localization of the protein in stroma and around the vessels in EuE and EcE (**Figures 4C–F**; **Supplementary Figures 6**-**8**). In line with qPCR data, TGFBI protein expression in EuE was also higher in the proliferative phase compared to secretory phase (**Supplementary Figure 9**). When we compared paired EuE and EcE in the secretory phase, TGFBI staining was stronger in the stroma of EcE, as what we observed in SC cultures. While TGFBI role in endometriosis in the tissues is less clear according to the results from our *ex vivo* experiments, its roles in fibrosis and angiogenesis cannot be underestimated. The immunoreaction around the vessels might indicate that the perivascular cell populations might be an important contributor to the increased levels of secreted TGFBI protein.

This study has several limitations, with the major limitation represented by the small sample size in our *in vitro* experiments. In case of protein kinase assay, we used previously developed in-house synthesized probes – thus, the examined set of targets was limited to PKAc, ROCK, Akt, and CK2. In case of transcriptome study, a relatively low number of DEGs in some groups can be explained by the interpatient variation within the groups which affected the statistical significance of comparisons. The number of paired tissue samples of EuE and EcE in the proliferative phase was limited and thus no statistically valid comparison regarding TGFBI expression levels in EcE between the proliferative and secretory phase can be drawn. For the same reason, we could not examine *TGFBI* mRNA expression in paired EuE and EcE. In addition, the presence of fibrotic areas in EcE complicated the quantitative analysis of TGFBI protein signal intensity between paired EuE and EcE in the secretory phase.

Nonetheless, within this study we showed hypoxia-mediated transcriptome changes characteristic to EuESCs and EcESCs and identified TGFBI as a potential target for further studies addressing therapeutic strategies for endometriosis.

## Data Availability

The mRNA sequencing raw data are available in online repository Gene Expression Omnibus (GEO) at https://www.ncbi.nlm.nih.gov/geo/ and can be accessed with GEO accession number GSE269530.
