# Supplementary material for: Hypoxic conditions affect transcriptome of endometrial stromal cells in endometriosis and promote TGFBI axis
Source: Front Endocrinol (Lausanne). 2024 Dec 18;15:1465393. doi: 10.3389/fendo.2024.1465393 (PMC11688238; doi:10.3389/fendo.2024.1465393)
Supplement: Supplementary file 1 [file DataSheet1.docx]

Supplementary Material

Hypoxic conditions affect transcriptome of endometrial stromal cells in endometriosis and promote TGFBI axis

Meruert Sarsenova^1,2,3^, Nageswara Rao Boggavarapu^2^, Keiu Kask^1,3^, Vijayachitra Modhukur^1,3^, Külli Samuel^3^, Helle Karro^1,4^, Kristina Gemzell-Danielsson^2^, Parameswaran Grace Luther Lalitkumar^2^, Andres Salumets^1,3,5,6^, Maire Peters^1,3^, Darja Lavogina^3,7,8^

*** Correspondence:** Andres Salumets, Division of Obstetrics and Gynaecology, Department of Clinical Science, Intervention and Technology (CLINTEC), Karolinska Institutet, and Karolinska University Hospital, 14152, Stockholm, Sweden. E-mail: andres.salumets@ki.se. https://orcid.org/0000-0002-1251-8160.

# Supplementary Figures


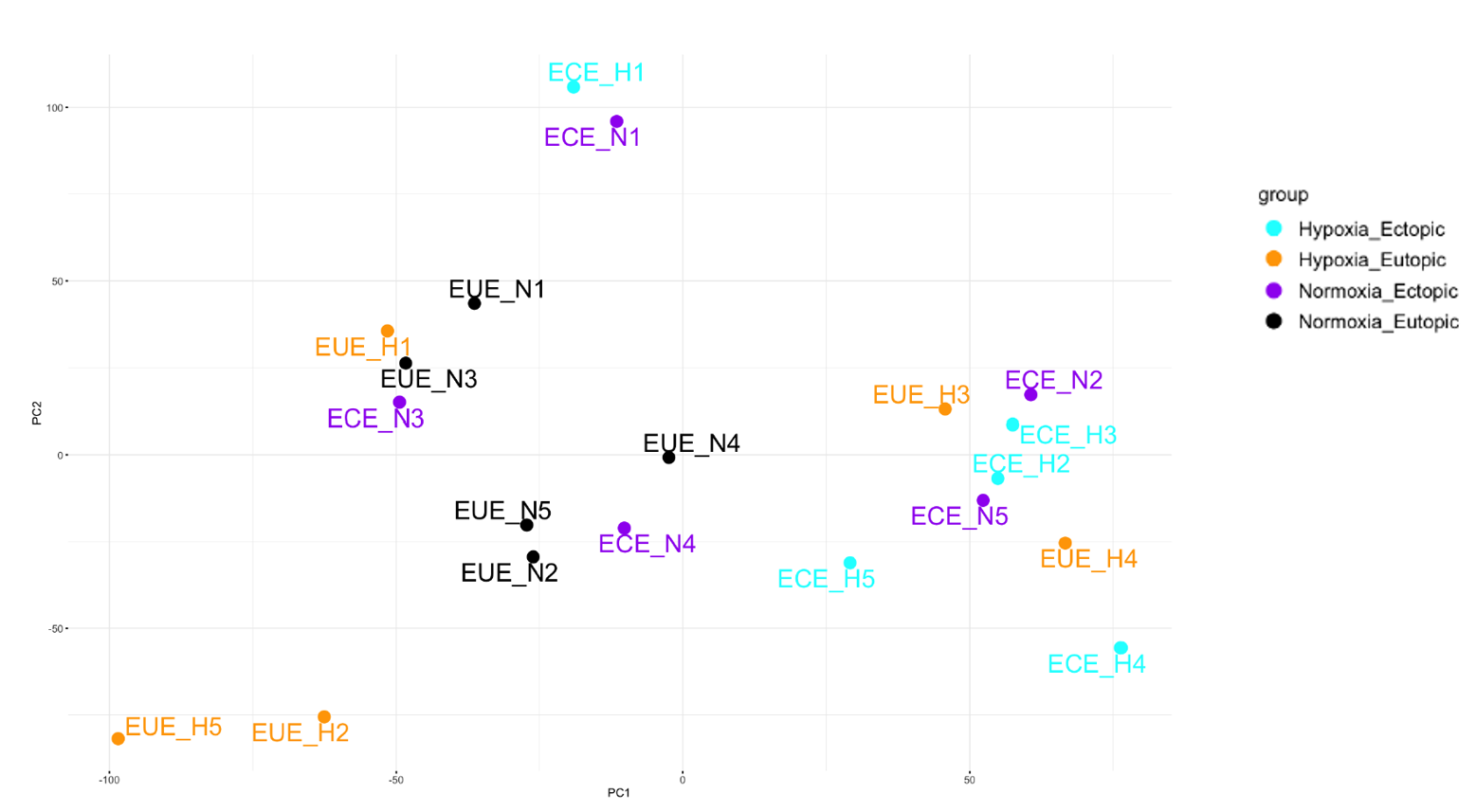


**Supplementary Figure 1.** PCA plot depicting the differences between the samples of cultured eutopic endometrial stromal cells (EuESCs, N = 5) and ectopic endometrial stromal cells (EcESCs, N = 5) exposed to normoxia or hypoxia. EUE_H1 - EUE_H5 – sample IDs corresponding to EuESCs exposed to hypoxia; EUE_N1 - EUE_N5 – sample IDs for EuESCs exposed to normoxia; ECE_H1 - ECE_H5 – sample IDs for EcESCs exposed to hypoxia; ECE_N1 - ECE_N5 – sample IDs for EcESCs exposed to normoxia; PCA - principal component analysis.


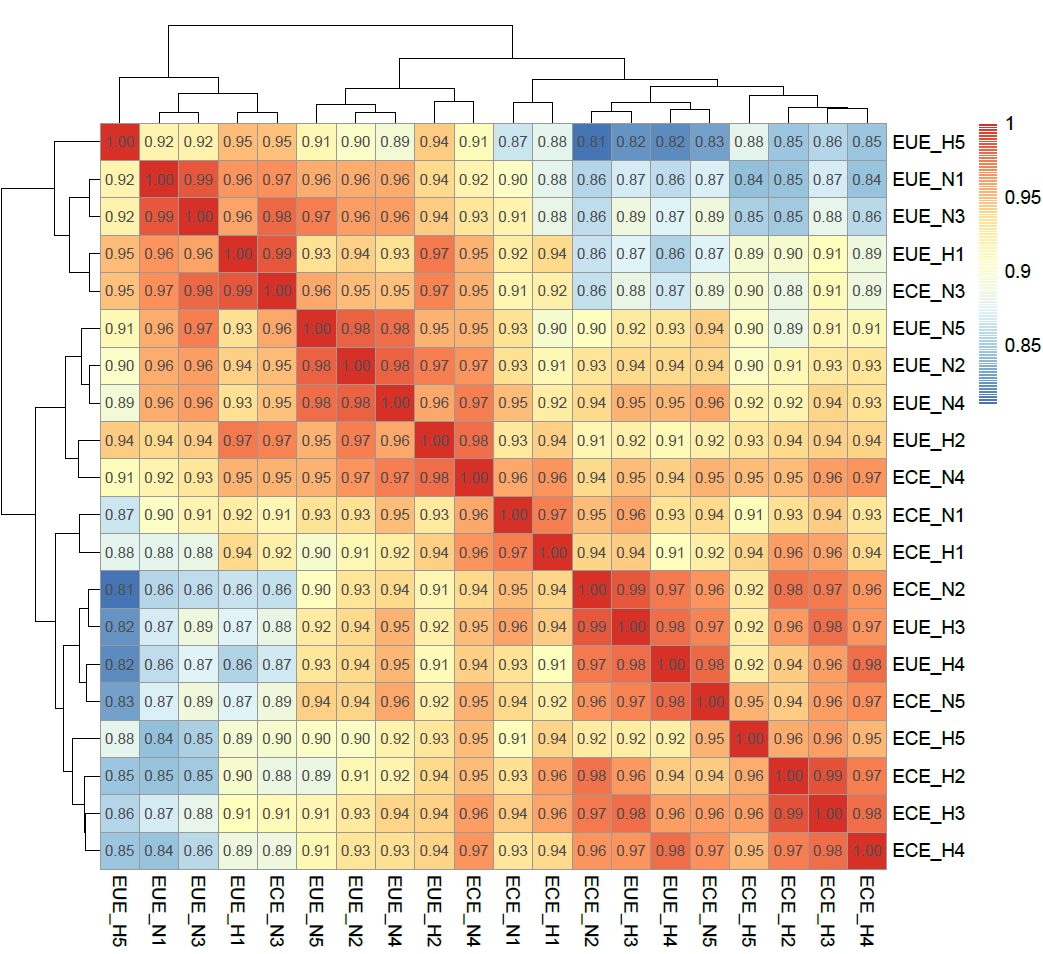


**Supplementary Figure 2.** A heatmap of Pearson correlation coefficient values of the samples of cultured eutopic endometrial stromal cells (EuESCs, N = 5) and ectopic endometrial stromal cells (EcESCs, N = 5) exposed to normoxia or hypoxia. EUE_H1 - EUE_H5 – sample IDs corresponding to EuESCs exposed to hypoxia; EUE_N1 - EUE_N5 – sample IDs for EuESCs exposed to normoxia; ECE_H1 - ECE_H5 – sample IDs for EcESCs exposed to hypoxia; ECE_N1 - ECE_N5 – sample IDs for EcESCs exposed to normoxia.


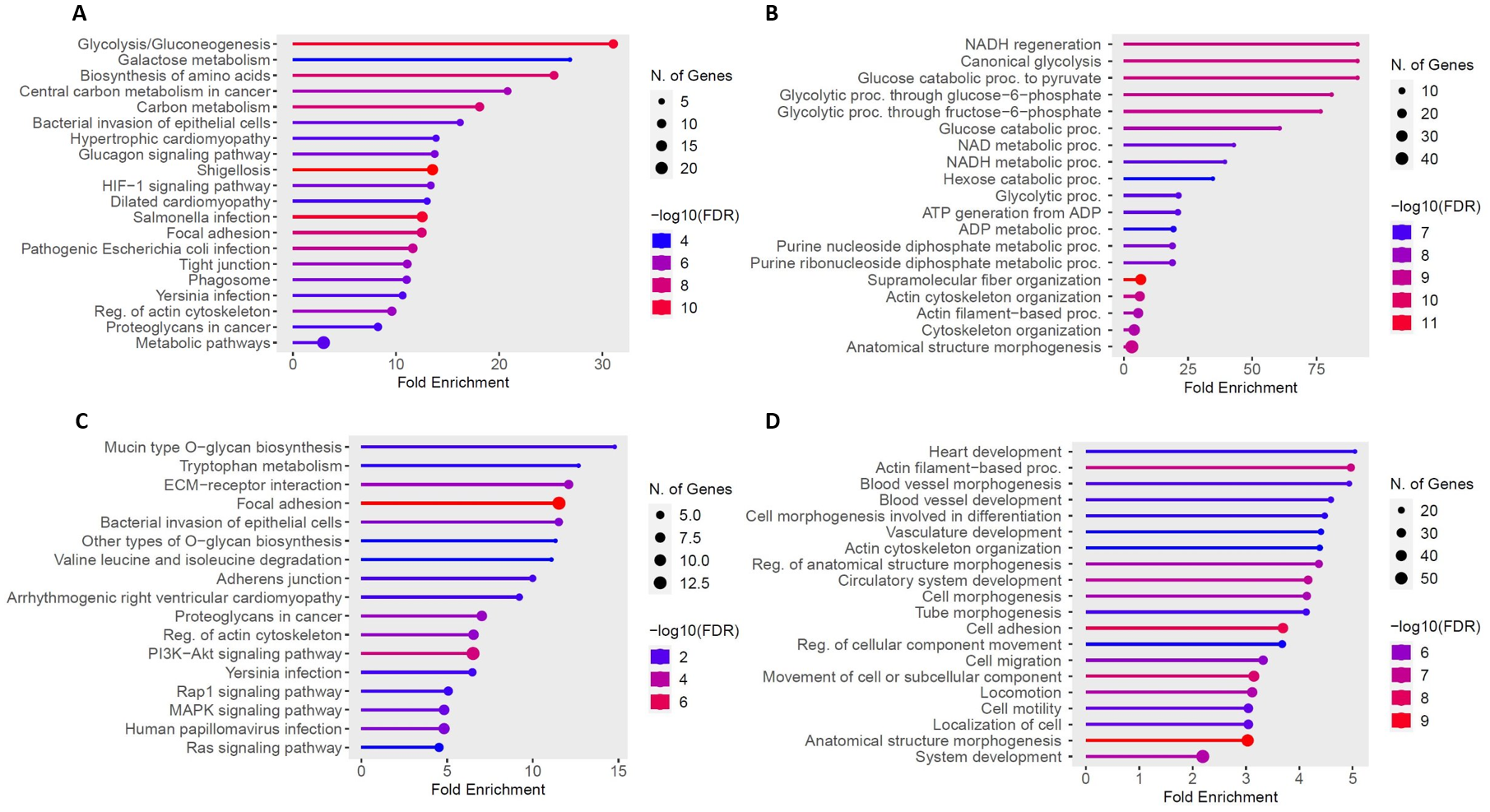


**
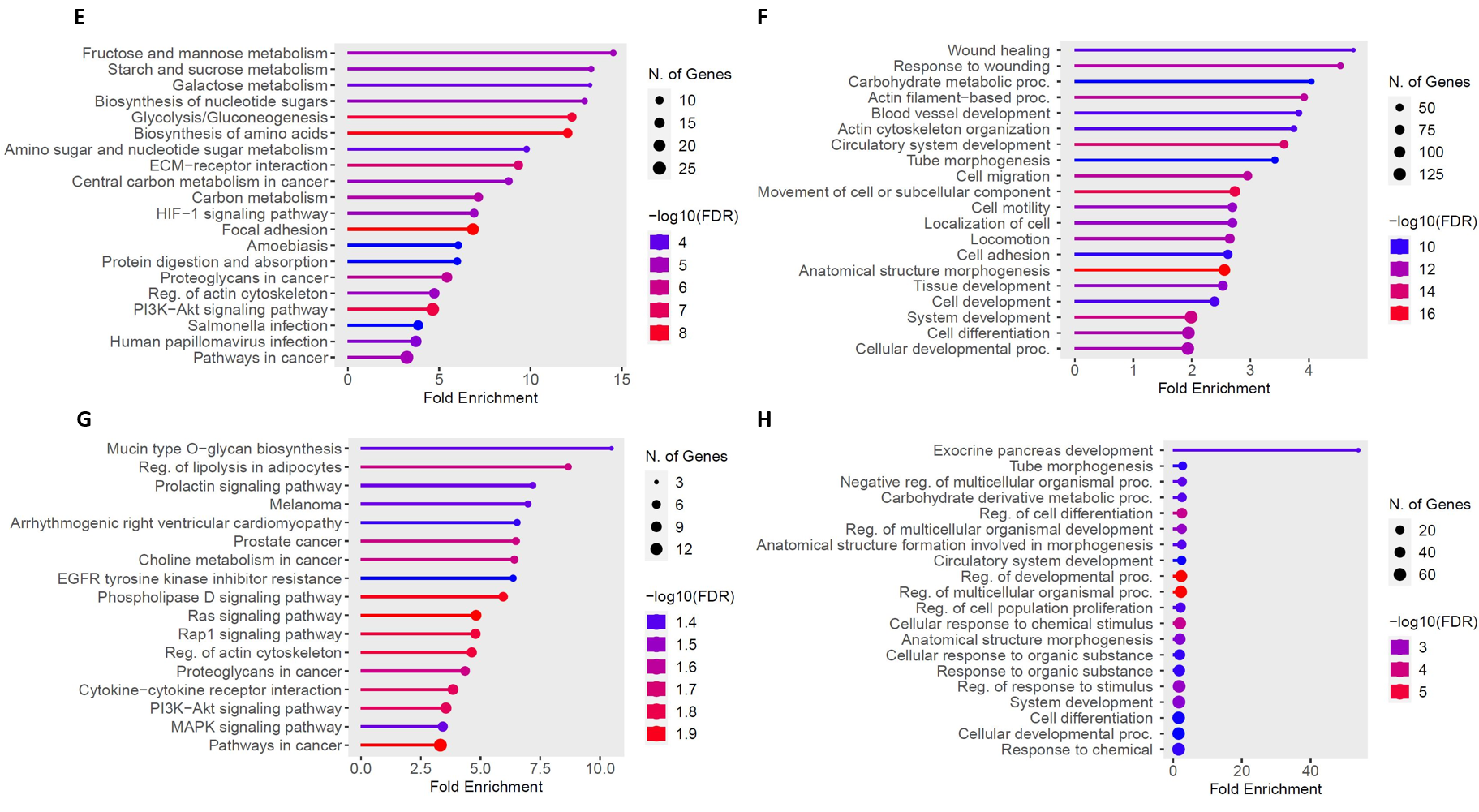
Supplementary Figure 3.** The top enriched pathways (KEGG, **A**, **C**, **E** and **G**) and biological processes (GO:BP, **B**, **D**, **F** and **H**) for different comparisons in transcriptome study. Up to 20 pathways and biological processes are presented for the following comparisons: EuESCs exposed to hypoxia vs normoxia **(A and** **B)**; EcESCs vs EuESCs exposed to normoxia **(C and** **D)**; EcESCs exposed to hypoxia vs EuESCs exposed to normoxia, upregulated genes’ enrichment **(E and F)**, and downregulated genes’ enrichment **(G and H)**. The analysis is based on statistically significant differentially expressed genes (DEGs), P_adj_ < 0.05. EuESCs – eutopic endometrial stromal cells; EcESCs – ectopic endometrial stromal cells.


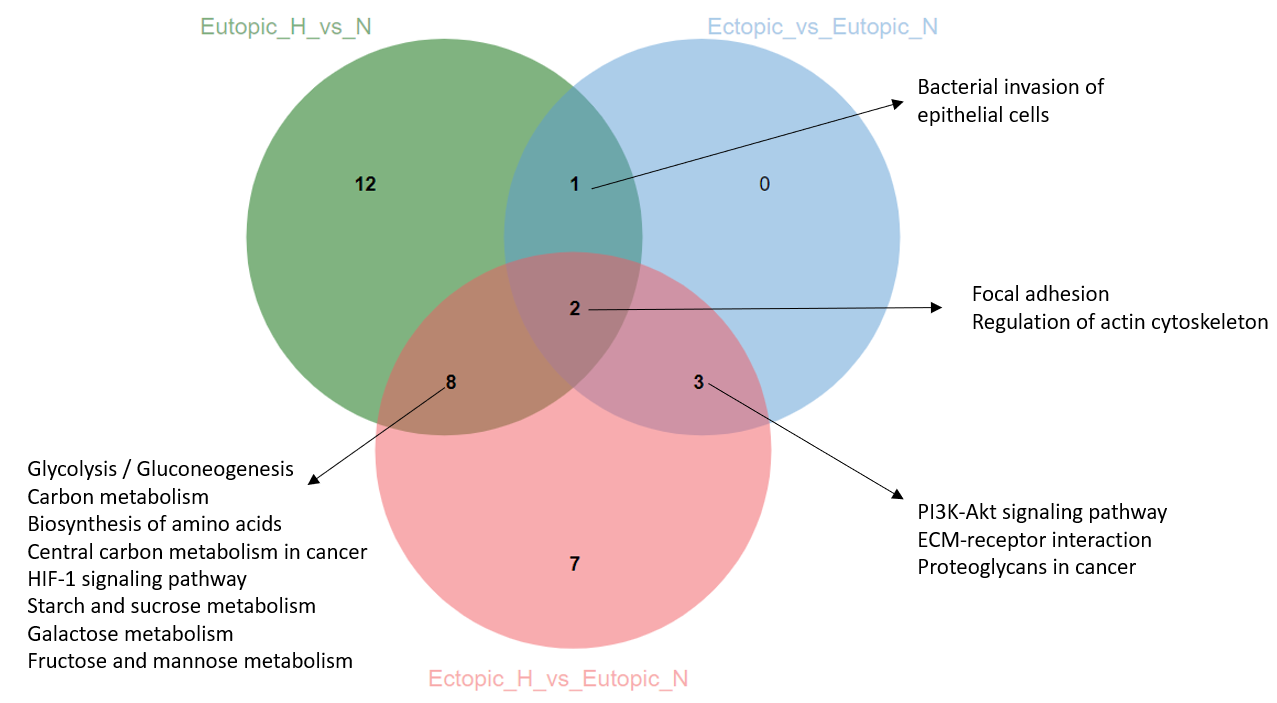


**Supplementary Figure 4.** The overlapped KEGG pathways between the groups: EuESCs exposed to hypoxia vs normoxia (Eutopic_H_vs_N), EcESCs vs EuESCs exposed to normoxia (Ectopic_vs_Eutopic_N), and EcESCs exposed to hypoxia vs EuESCs exposed to normoxia (upregulated genes, Ectopic_H_vs_Eutopic_N). The analysis of KEGG pathways is based on statistically significant differentially expressed genes (DEGs), P_adj_ < 0.05. EuESCs – eutopic endometrial stromal cells; EcESCs – ectopic endometrial stromal cells.


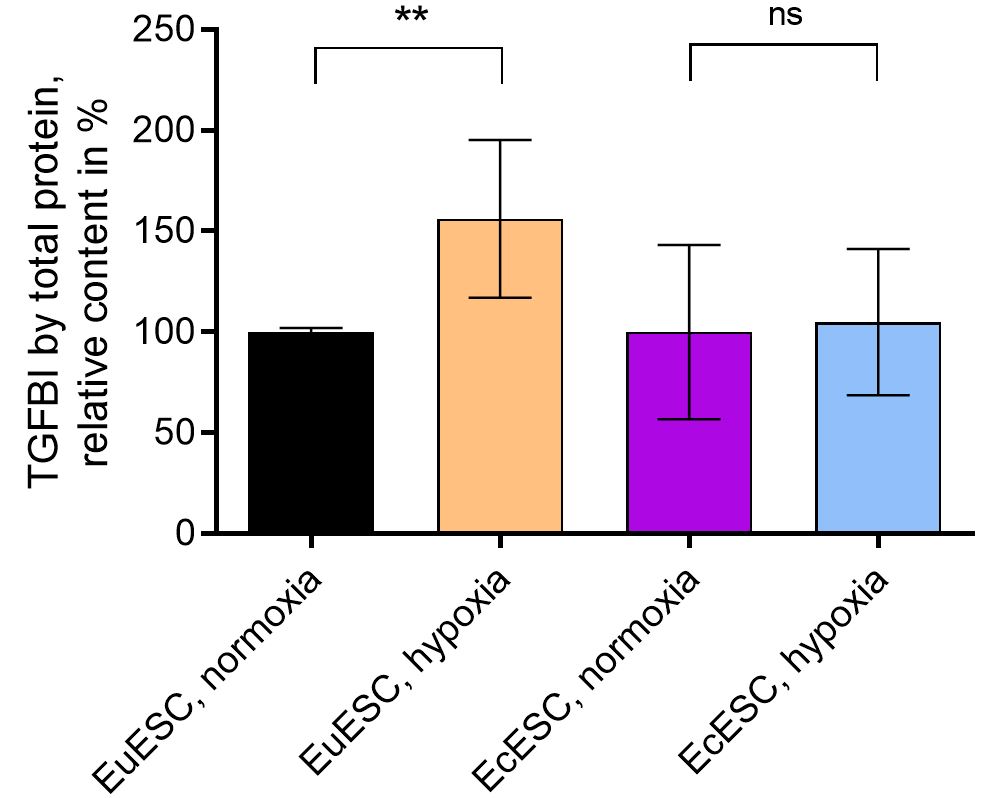


**Supplementary Figure 5.** Secreted TGFBI protein levels in spent cell culture media relative to the total protein content in the corresponding cell lysates. For each patient, the data was normalized to the relative TGFBI content measured for the EuESCs or EcESCs incubated in normoxia (= 100%). Each column shows the mean ± standard deviation for samples obtained from 5 different patients. Arrows and asterisks indicate pairwise comparisons (t-test with Welch’s correction): ** P ≤ 0.01, ns – not significant. EcESCs – ectopic endometrial stromal cells; EuESCs - eutopic endometrial stromal cells.


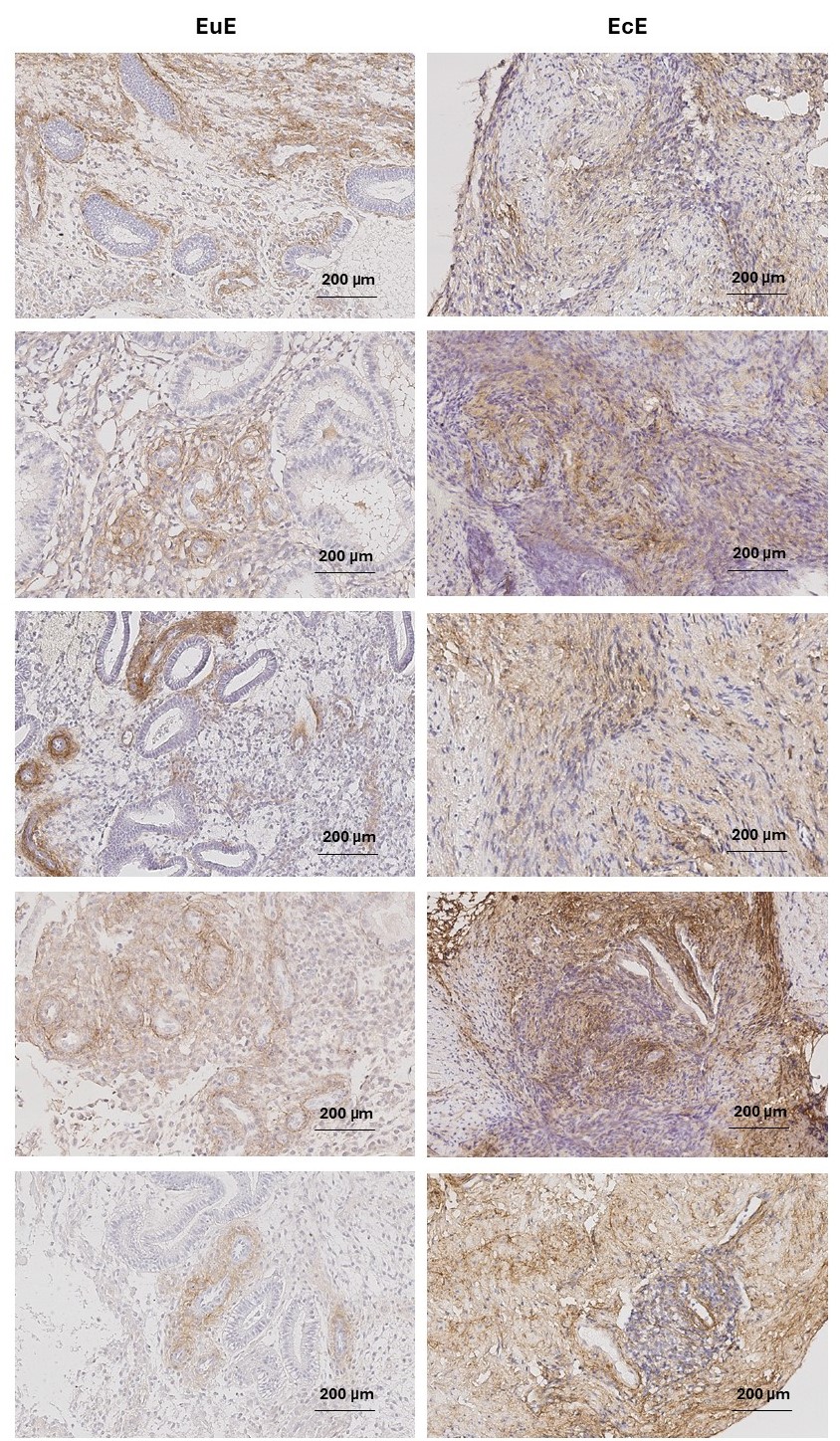


**Supplementary Figure 6.** Representative images of TGFBI protein staining of paired eutopic (EuE, left column) and corresponding ectopic (EcE, right column) endometrium from patients with endometriosis (N = 5) in the secretory phase of the menstrual cycle, showing the expression of TGFBI protein in stroma of eutopic endometrium and around the vessels. Scale bar 200 μm.


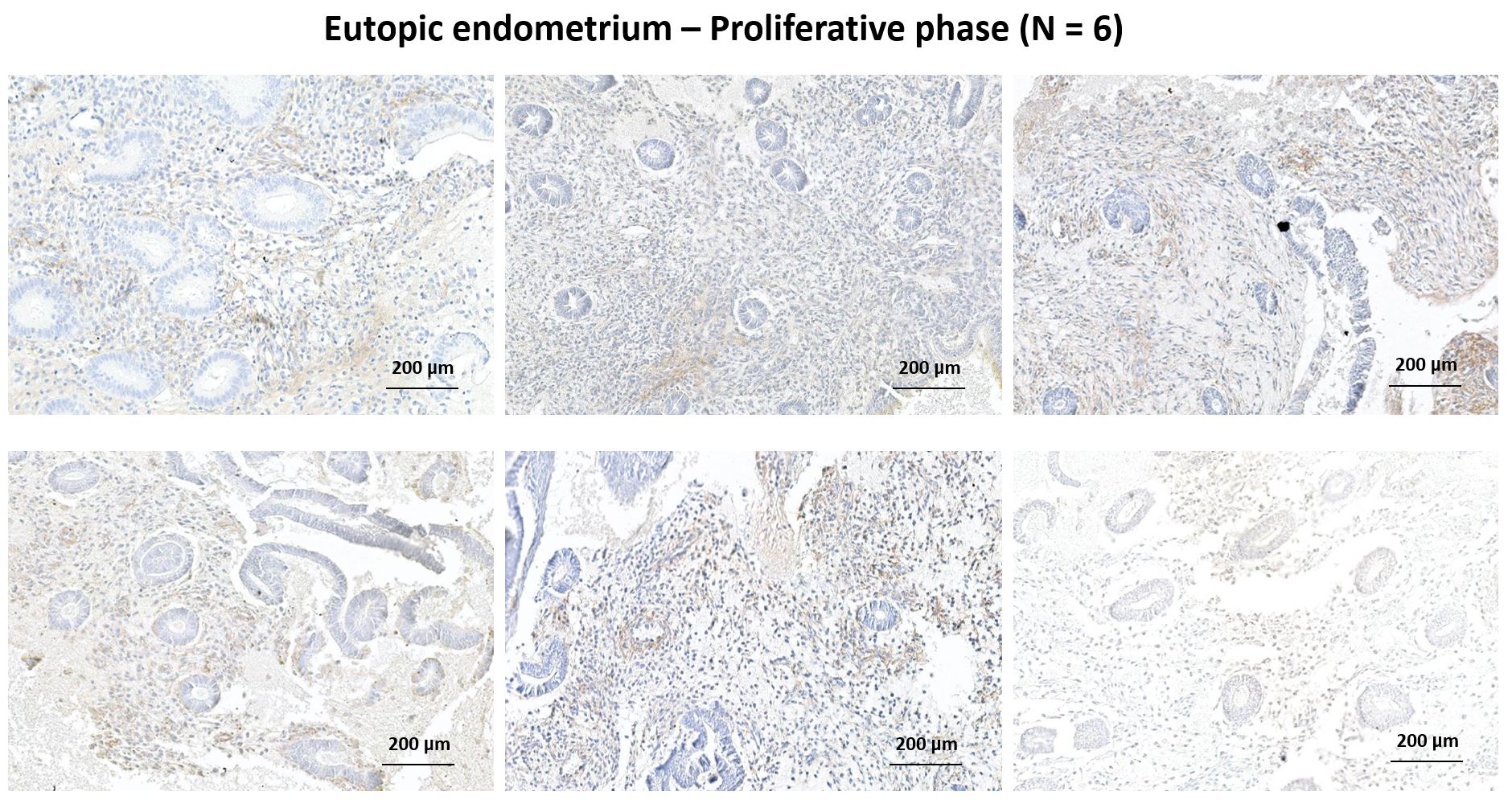
 **Supplementary Figure 7.** Representative images of TGFBI protein staining of eutopic endometrium from patients with endometriosis in proliferative phase of the menstrual cycle (N = 6), showing the expression of TGFBI protein in stroma of eutopic endometrium and around the vessels. Scale bar 200 μm.


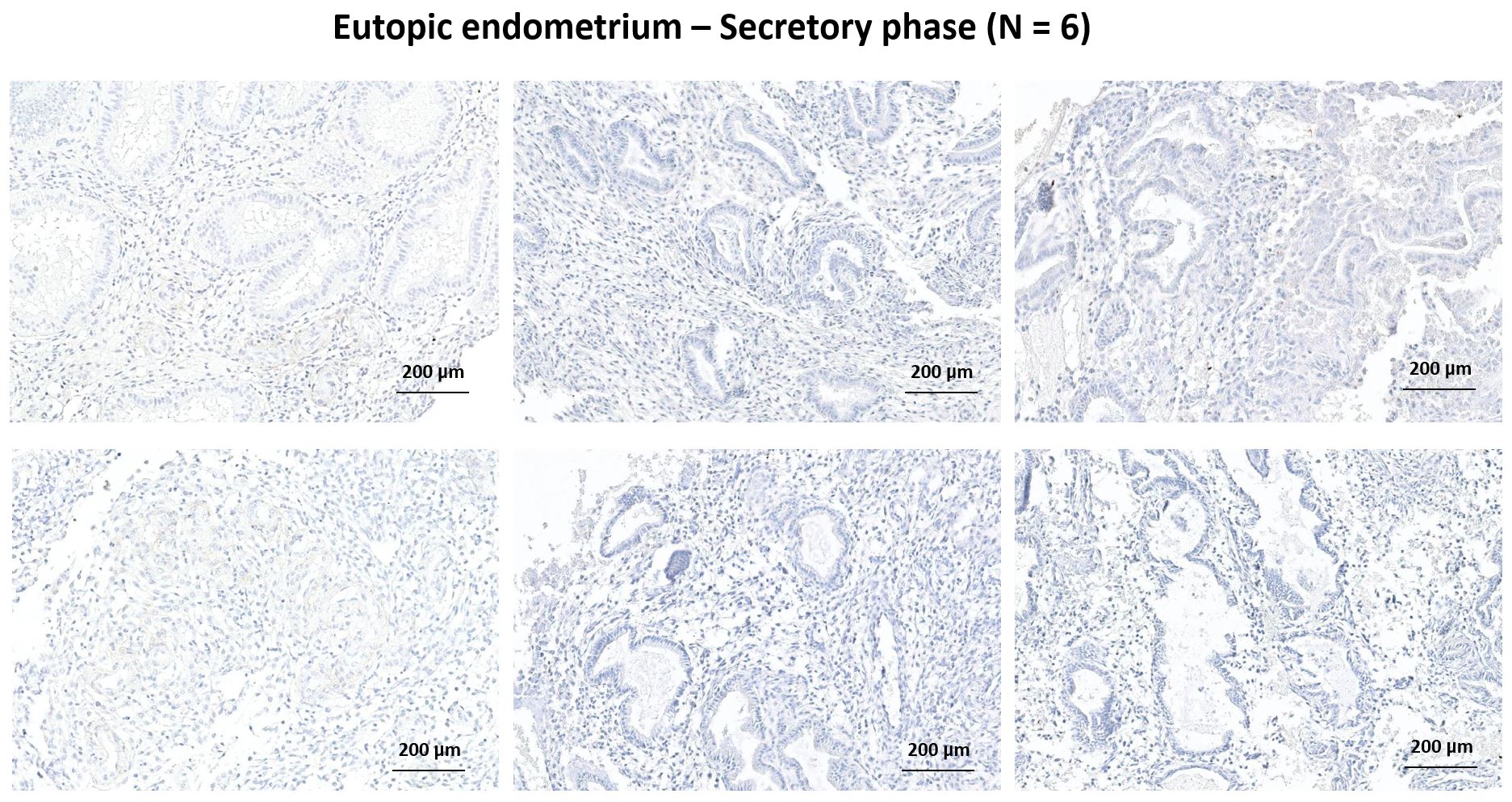


**Supplementary Figure 8.** Representative images of TGFBI protein staining of eutopic endometrium from patients with endometriosis in secretory phase of the menstrual cycle (N = 6), showing weak expression of TGFBI protein in stroma of eutopic endometrium. Scale bar 200 μm.


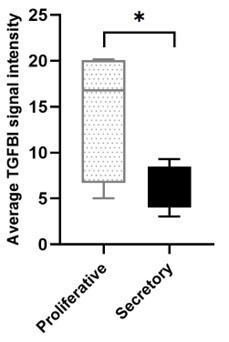


**Supplementary Figure 9.** TGFBI protein signal intensity in eutopic endometrium from patients with endometriosis in proliferative vs secretory phase of the menstrual cycle (N = 6 and N = 11, respectively), * P value < 0.05.

# Supplementary Tables*

**Supplementary Table S1.** Statistically significant DEGs in 5 comparison groups. Eutopic endometrial stromal cells (EuESCs), ectopic endometrial stromal cells (EcESCs). Genes with an absolute log_2_ fold change |log_2_FC| > 0.5 and FDR-adjusted P-value (P_adj_) < 0.05 were considered as differentially expressed.

**Supplementary Table 2.** Gene set enrichment analysis (g:Profiler online tool) representing statistically significantly enriched terms, including gene ontology, KEGG and Reactome pathway analyses. The table includes representative enriched terms and representative DEGs in brackets. Abbreviations: DE – differential expression, GO – gene ontology, MF – molecular function, BP – biological process, CC – cell compartment, REAC – Reactome, EuESCs – eutopic endometrial stromal cells, EcESCs – ectopic endometrial stromal cells.

*These Supplementary Tables are attached separately in a single Excel document due to the large size of the tables.
